# Robotic high anterior resection for rectal cancer with hand-sewn Gambee anastomosis after metal stent placement in a colorectal malignant stricture: A case report

**DOI:** 10.1016/j.ijscr.2024.109383

**Published:** 2024-02-10

**Authors:** Toshiyuki Suzuki, Akiyo Matsumoto, Takahiko Akao, Hiroshi Matsumoto

**Affiliations:** Department of Surgery, Hanyu General Hospital, Hanyushi Saitama 348-8505, Japan

**Keywords:** Robotic-assisted surgery, Gambee anastomosis, Colorectal malignant stricture, Metal stent placement, Case report, Double stapling technique

## Abstract

**Introduction:**

Robot-assisted surgery is increasingly deployed in colorectal surgery, and decompression surgery using a stent is considered a standard treatment for malignant stenosis of the large intestine. Surgery after stent placement is also frequently performed. However, the anastomosis method remains controversial.

**Presentation of case:**

A 75-year-old woman visited our hospital's internal medicine department with chief complaints of bloody stool and constipation for the past year and colonoscopy was scheduled. After taking laxatives to prepare for treatment, abdominal pain was noticed and an emergency request was made. A diagnosis of colorectal malignant stricture and rectosigmoid junction cancer was made and a stent was placed during emergency colonoscopy. After intestinal decompression, a diagnosis of rectosigmoid junction cancer (UICC 8th; T3N0M0 Stage IIa) was rendered and robotic-assisted high anterior resection of the rectum and lymph node D3 dissection were performed. Reconstruction was performed using Gambee anastomosis outside the body cavity. The postoperative course was uneventful.

**Discussion:**

The double stapling technique is simple, but in this case, the obstructed intestinal tract was swollen. Meanwhile, Gambee anastomosis, which allows adjustment of tightness, was considered effective.

**Conclusion:**

Gambee anastomosis is a valid option when robot-assisted rectal resection is performed after intestinal decompression with stent placement for malignant stricture of the rectosigmoid junction. It is important to select a hand-sewn or mechanical anastomosis by considering the condition of the organ to be anastomosed and the site of the anastomosis.

## Highlights

• Gambee anastomosis is a valid option for rectosigmoid junction stricture.
• Suture failure can occur in swollen and obstructed intestinal tracts.
• The hand-sewn or mechanical anastomosis should match the condition of the organ.

## 1 Introduction

Robotic-assisted surgery using a surgical robot has important features, such as forceps with a wide range of motion, anti-shake mechanism, high-resolution three-dimensional imaging, and functions, including image stabilization and motion scaling. [1] Therefore, robot-assisted surgery is minimally invasive, and it increases the surgeon's freedom to create the desired shape, making it possible to perform accurate and precise surgery. Therefore, in the field of colorectal cancer, surgery for colon and rectal cancer is rapidly becoming popular. [2,3]

It has been reported that 15 %–30 % of colorectal cancers are accompanied by large intestinal obstruction [ 4]. Recently, self-expanding metal stent (SEMS) insertion in the obstructed colon emerged as a minimally invasive and relatively simple procedure providing an effective first-line treatment for the relief of acute malignant obstruction symptoms and serving either as a preoperative or “bridge to surgery” procedure. [5] As a subsequent step, it is common to perform radical surgery after improving the patient's general condition. Although the obstruction is improved by SEMS placement, it is believed that the large intestine remains affected by the expansion. Therefore, it is important to choose between hand-sewn and instrument-assisted double stapling technique (DST) anastomosis during radical surgery. However, the option for definitive surgical anastomosis after metal stent placement in colorectal malignant strictures remains controversial.

In this study, we report an adult case of robotic high anterior resection for rectosigmoid junction cancer with hand-sewn Gambee anastomosis after metal stent placement in a colorectal malignant stricture. This report aimed to provide information on the safety and usefulness of robotic high anterior resection for rectosigmoid junction cancer with hand-sewn Gambee anastomosis after metal stent placement in colorectal malignant strictures under robot-assisted surgery. This case report was reported in line with the SCARE 2023 criteria [6].

## 2 Presentation of the case

**Fig. 1:**
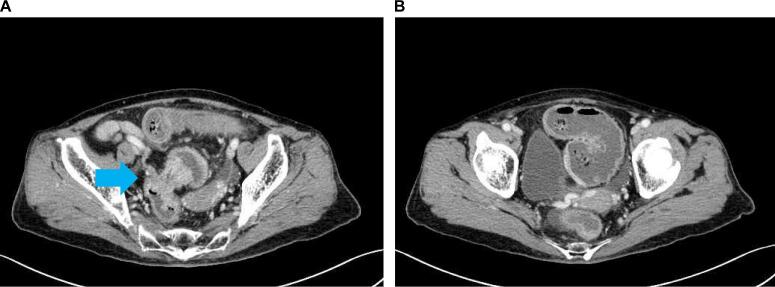
Computed tomography findings. In the rectosigmoid junction, thickening of the wall with contrast enhancement (arrow) and dilatation of the colon proximal to the sigmoid colon were observed.

**Fig. 2:**
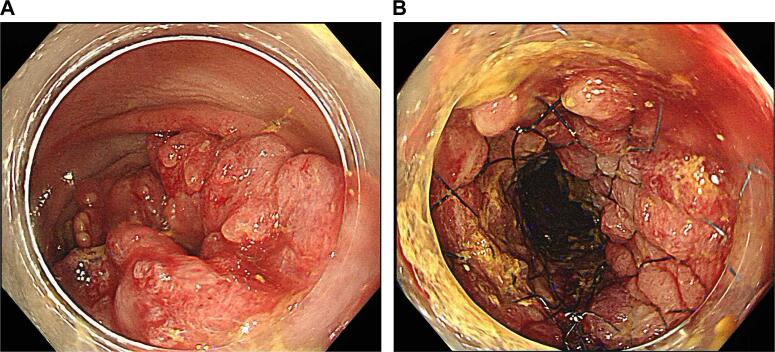
Lower gastrointestinal endoscopy findings. (a) Circumferential type 3 rectosigmoid junction cancer was observed. (b) After self-expanding metal stent placement.

**Fig. 3:**
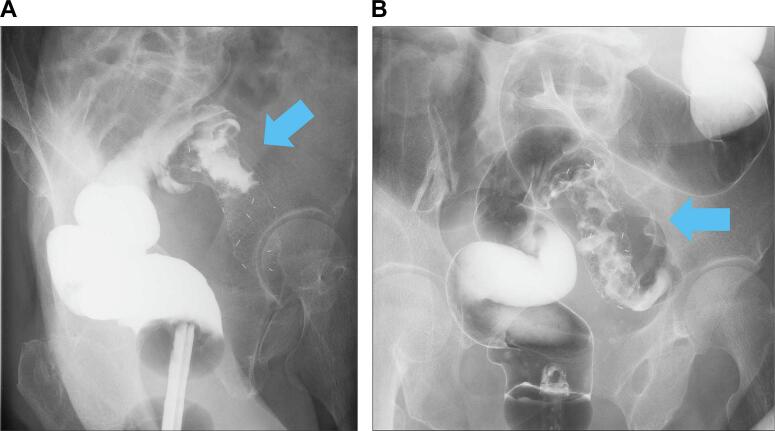
Double-contrast gastrografin enema findings. Gastrografin enema revealed a tumor in the circumferential wall of the sigmoid colon extending from the rectosigmoid junction. The arrows point to the tumor. (a) Sagittal plane. (b) Coronal plane.

**Fig. 4:**
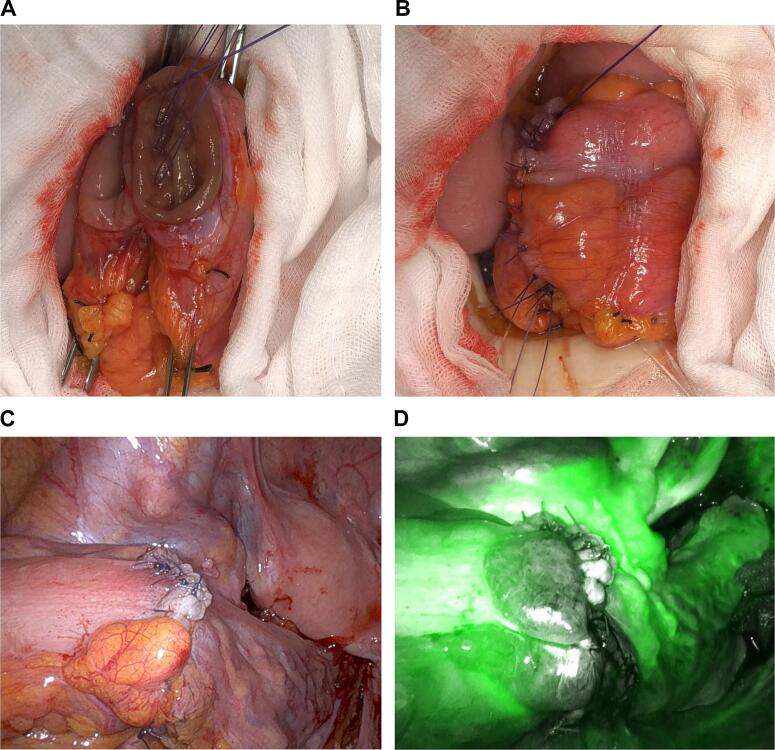
Intraoperative findings. Gambee anastomosis using 4–0 Vicryl during extracorporeal reconstruction. (a) Posterior wall sutures. (b) Anterior wall sutures. Intraoperative laparoscopic findings after reconstruction. (c) Reconstruction department. (d) Post-anastomotic indocyanine green perfusion assessment. (For interpretation of the references to colour in this figure legend, the reader is referred to the web version of this article.)

The patient was a 75-year-old woman who presented with bloody stool and constipation. She was rushed to the hospital because she developed abdominal pain after taking laxatives as a preparatory treatment for colonoscopy. Computed tomography revealed a colorectal malignant stricture and rectosigmoid junction cancer (Fig. 1a, b). No distant or lymph node metastases were observed. Colonoscopy revealed circumferential T3 rectosigmoid junction cancer, and an SEMS was placed during emergency colonoscopy (Fig. 2a, b). The tumor was located 15 cm from the anal verge. After intestinal decompression, a diagnosis of rectosigmoid junction cancer (T3N0M0 Stage IIa; UICC 8th edition) was made based on the findings of the imaging tests (Fig. 3a, b). The patient was malnourished at the time of examination; however, her nutritional status improved following oral intake and fluid replacement after stent placement. As diabetes mellitus was confirmed, radical surgery was performed 1 month after her blood sugar was controlled. We performed high anterior resection of the rectum and lymph node D3 dissection using the da Vinci Xi surgical system (Intuitive Surgical Inc., Sunnyvale, CA, USA). The patient was placed right side down in the Trendelenburg position. One 12-mm camera port, three 8-mm robotic ports, one pneumoperitoneum port, and one laparoscopic assistant port were placed. Medial-to-lateral dissection of the sigmoidal mesocolon with high ligation of the inferior mesenteric artery was performed and the sigmoid colon was separated from the lateral attachment. The sigmoid colon appeared to be longer than normal. Total mesorectal excision using a nerve-preserving technique was achieved. An additional umbilical incision was made at the camera port site, and the rectosigmoid tumor was exposed outside the abdominal cavity using the Alexis® wound retractor system. Next, more than 10 cm of the rectosigmoid proximal to the tumor were transected extracorporeally. Extracorporeal reconstruction was performed using Gambee anastomosis with 4–0 Vicryl (Fig. 4a, b). Intestinal blood flow was assessed using the indocyanine green fluorescence method for the oral side of the intestine and no problems were detected (Fig. 4c, d). A pleated drain was placed at the anastomotic site to check for leakage. The total operative time was 273 min, including a robotic time of 143 min and a reconstruction time of 42 min, and the blood loss was 11 mL. Pathological examination revealed pT3, pN0, cM0, and pStage IIA disease and confirmed R0 resection. The postoperative course was uneventful with no anastomotic leakage or other complications. Neither recurrence nor problems with defecation have been noted in the 6 months since surgery.

## 3 Discussion

Gambee anastomosis, which is a hand-sewn suture, is useful in cases such as the present case in which intestinal edema remains after stent placement because of large intestine obstruction. The advantages of Gambee anastomosis are that the suturing force for each stitch can be finely adjusted and the suture width can be adjusted. During intestinal anastomosis, the intestinal tract should be handled cautiously. When threading the intestinal tract, the serosa is held firmly, and the thread is threaded through the serosa muscle layer. In addition, when suturing, the threads should be tightened slowly over time rather than suddenly. In this manner, in hand-sewn Gambee anastomosis, sutures that match the condition of the intestinal tract can be made. Conversely, in edematous intestinal tracts, instrumental anastomoses such as DST anastomoses can tear if sutured with constant force. Therefore, DST anastomosis is not suitable in cases of intestinal edema such as that caused by large bowel obstruction because of the risk of suture failure. Furthermore, even if a stent is placed to alleviate the structure of the large intestine obstruction caused by a tumor, it is considered that some damage to the intestine will remain during intestinal expansion, and we believe that there is a considerable risk of suture failure due to DST anastomosis.

Colorectal anastomosis with DST is a common and widely used procedure with several advantages, including technical ease and a low risk of contamination [ [7], [8], [9], [10]]. Gambee anastomosis is a slower and more laborious procedure than DST anastomosis. However, it is a critical skill for general surgeons. Currently, instrumental anastomosis is the mainstream approach, but there are many cases in which hand-sewn anastomosis is effective, such as the present case. It has been reported that 15 %–30 % of colorectal cancers are accompanied by large intestine obstruction [ 4], and in many cases, the tumor is removed after stent placement. Hand-sewn anastomosis is effective, as in this case, and the technique can reduce the risk of suture failure. Therefore, although instrumental anastomosis is the mainstream technique, it is important to acquire hand-sewn suturing techniques such as Gambee anastomosis. Furthermore, in this case, anastomosis took 42 min, indicating that this procedure can be performed quickly.

Hand-sewn anastomoses might have a higher risk of surgical site infection (SSI) than DST anastomoses [ 7]. To reduce the risk of SSI, the Alexis® wound retractor system was used in this case. Capolupo et al. reported that SSI rates were significantly reduced by use of the Alexis® wound retractor system. [11] Furthermore, in this case, the wound was thoroughly washed with physiological saline immediately before skin closure, and then the skin was closed. Thus, it is believed that SSIs can be avoided by taking preventative measures.

## 4 Conclusion

It is important to perform hand-sewn or mechanical anastomosis by considering the condition of the organ being anastomosed and the site of the anastomosis. Hand-sewn Gambee anastomosis is a valid option when robot-assisted rectal resection is performed after intestinal decompression with stent placement for malignant stricture of the rectosigmoid junction.

## Consent

Written informed consent was obtained from the patient for publication of this case report and the accompanying images. A copy of the written consent is available for review by the Editor-in-Chief of this journal on request.

## Ethical approval

The IRB/Ethics Committee of Hanyu General Hospital ruled that approval was not required for this study.

## Funding

None.

## Author contribution

Toshiyuki Suzuki: Conceptualization, Methodology, Software, Validation, Formal analysis, Investigation, Resources, Writing—Original Draft, Visualization, Project administration, Funding acquisition.

Akiyo Matsumoto: Data curation and Supervision.

Takahiko Akao: Data curation.

Hiroshi Matsumoto: Writing—Review & Editing.

## Guarantor

Toshiyuki Suzuki.

## Research registration number

1. Name of the registry: jRCT: Japan Registry of Clinical Trials.
2. Unique identifying number or registration ID: In process.
3. Hyperlink to your specific registration (must be publicly accessible and will be checked): https://jrct.niph.go.jp.

## Conflict of interest statement

None.

## Data Availability

The datasets used and/or analysed during the current study are available from the corresponding author on reasonable request.
